# Retinal Nerve and Vascular Changes in Prediabetes

**DOI:** 10.3389/fmed.2022.777646

**Published:** 2022-02-17

**Authors:** Rui Ping Peng, Zi Qian Zhu, Hong Yi Shen, Hong Mei Lin, Lei Zhong, Si Qi Song, Tian Liu, Shi Qi Ling

**Affiliations:** ^1^Department of Ophthalmology, The Third Affiliated Hospital, Sun Yat-sen University, Guangzhou, China; ^2^Health Management Center, The Third Affiliated Hospital of Sun Yat-sen University, Guangzhou, China; ^3^Department of Ophthalmology, Guangzhou Women and Children's Medical Center, Guangzhou Medical University, Guangzhou, China

**Keywords:** prediabetes, nerve fiber layer, microvascular injury, impaired fasting glucose, impaired glucose tolerance (IGT)

## Abstract

**Objective:**

This study aimed to observe vascular and neuroretinal alterations in people with prediabetes [impaired fasting glucose (IFG) and impaired glucose tolerance (IGT)] and normal glucose metabolism.

**Methods:**

A total of 21 patients with prediabetes (42 eyes) and 20 healthy controls (40 eyes) participated in our study. All patients underwent a complete eye examination [including fundus fluorescein angiography (FFA) and optical coherence tomography (OCT)] and a related general examination (complete biochemical analysis, routine blood tests, and glycosylated hemoglobin).

**Results:**

On FFA, no patients in either group showed any microvascular alterations. The total peripapillary retinal nerve fiber layer (pRNFL) in the prediabetic group was significantly thinner than that in the healthy control group (*p* < 0.0001). Only the temporal pRNFL thickness was significantly less in patients with prediabetes compared to the normal people. There was no significant difference in the thickness of retina in the range of 1 mm diameter of macular fovea (*p* = 0.286), but in the prediabetic group, the macular retinal thickness within the diameter of 6 mm in nasal side (*p* < 0.0001), superior side (*p* < 0.0001), temporal side (*p* = 0.008), and inferior side (*p* = 0.001) were lower than that in the control group.

**Conclusion:**

In the prediabetic group, there was no microvascular alterations, but the total pRNFL and the temporal pRNFL was significantly thinner, and the macular retinal thickness within the diameter of 6 mm in the nasal, temporal, and inferior side were lower than that in the healthy control group. These data confirm neuroretinal alterations in prediabetes prior to microvascular injury.

## Introduction

Diabetes presents a heavy medical burden and increasing challenges for clinicians, both at present and in the coming decades. The prevalence of elevated blood glucose levels in populations in both developed and developing countries is rising rapidly, and the absolute number of patients with prediabetes is expected to increase by approximately one-third by the middle of this century (1). Increasing research has indicated that diabetic retinopathy (DR) is a multitissue lesion with neural and vascular interactions. The retina is rich in vascular and neural tissue, with neurons, glial cells, and the vascular system forming a whole interdependent retinal neurovascular unit. Our previous study showed that early diabetes was associated with potential retinal nerve damage (2). Furthermore, it is not clear whether retinal nerve damage and microangiopathy are present in prediabetes.

Prediabetes is a precursor to diabetes, which eventually leads to the development of diabetes in most cases. Prediabetes can be classified as impaired fasting glucose (IFG) or impaired glucose tolerance (IGT). Both IFG and IGT are risk factors for type 2 diabetes. The risk is greater when IFG and IGT occur together (3). The harm associated with the intermediate hyperglycemic state between normoglycemia and diabetes in prediabetes are well established, and the relationship between retinal neuropathy and microvascular damage is our focus of attention.

In our study, we aimed to observe whether there are differences in the retinal microvasculature and retinal nerves between prediabetic and normoglycemic populations and to analyze the correlation between systemic conditions such as BMI and levels of lipids, glycated hemoglobin, uric acid, and urea nitrogen and the observed differences.

## Materials and Methods

### Study Design and Setting

In this prospective study, 21 prediabetic patients and 20 healthy controls were recruited from the Third Affiliated Hospital of Sun Yat-sen University from January 2020 to January 2021. All prediabetes patients were diagnosed in the endocrinology department of the same hospital. This study was approved by the Ethics Committee of the Third Affiliated Hospital of Sun Yat-sen University [Guangzhou, (2018)02-009-01], and written informed consent was obtained from all participants in accordance with the Declaration of Helsinki.

### Selection of Participants and Treatment

The inclusion criteria for patients in this study were as follows: all prediabetes patients found to have abnormal glucose regulatory function, including IGT or IFG. IGT was defined as a two-hour oral glucose tolerance test (2 h OGTT) result higher than 7.8 mmol/L and lower than 11.1 mmol/L, and fasting blood glucose lower than 7.0 mmol/L. IFG refers to fasting glucose higher than 6.1 mmol/L and lower than 7.0 mmol/L, and blood glucose load lower than 7.8 mmol/L 2 h afterward. The exclusion criteria for all participants were any other diseases that could cause retinal and neurological damage, such as glaucoma with spherical equivalent more than plus or minus 2 diopters, optic neuropathy, age-related macular degeneration, retinal and choroidal disease, and retinal artery/vein occlusion; individuals with severe refractive media turbidity disease, such as cataracts; and individuals with hypertension, hemopathy, neuropathy, and other systemic diseases.

Prediabetes patients matched in age (*p* = 0.15), gender (*p* = 0.99), best corrected visual activity (BCVA, *p* = 0.09), intraocular pressure (IOP, *p* = 0.36), spherical equivalent (*p* = 0.24), and axis length (*p* = 0.44) served as healthy controls.

### Clinical Examinations and Laboratory Analyses

All participants underwent detailed laboratory testing, including fasting blood glucose (FBG), two-hour oral glucose tolerance test (2 h OGTT), routine blood, urine, and stool tests, blood biochemical indexes, and blood gas analysis.

**Figure 1:**
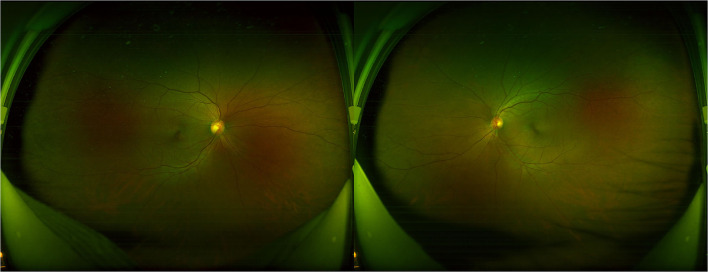
The wide-field fundus photograph shows no obvious abnormality in a prediabetes patient.

**Figure 2:**
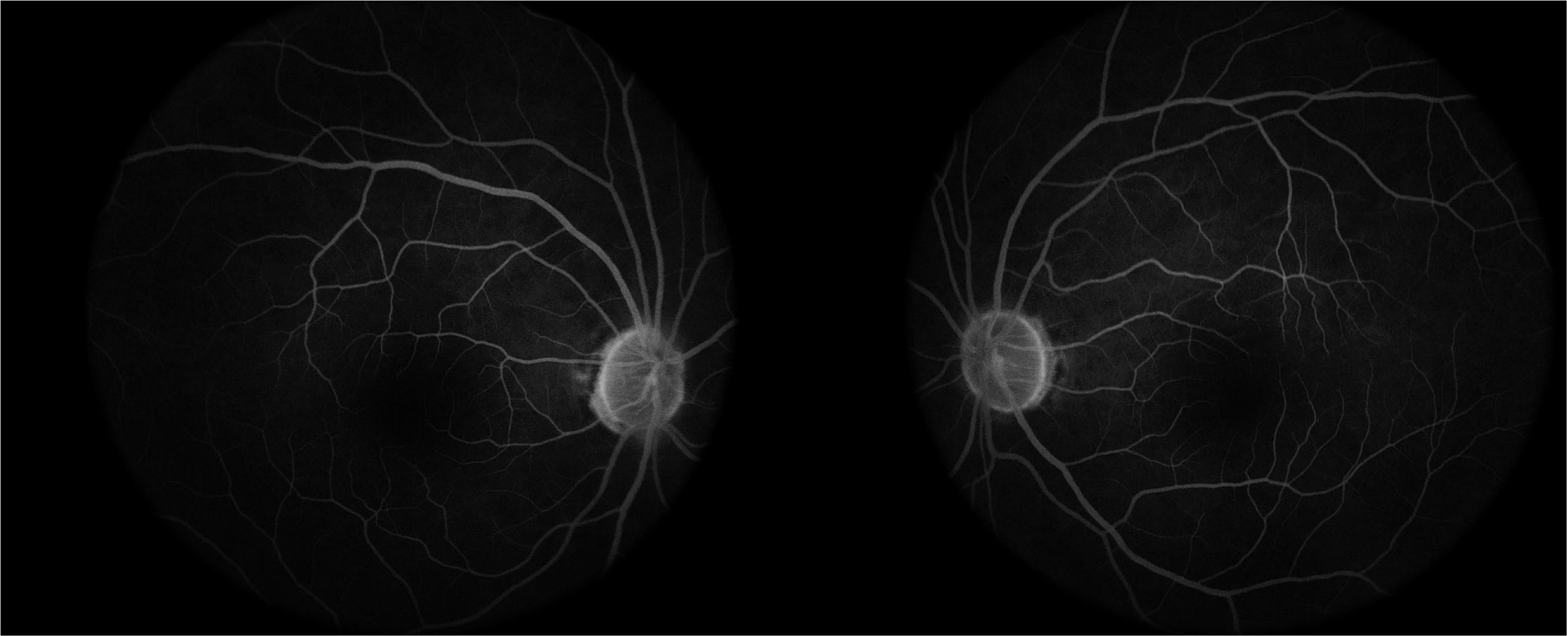
The fundus fluorescein angiography (FFA) examination of a prediabetes patient shows no obvious abnormality.

**Figure 3:**
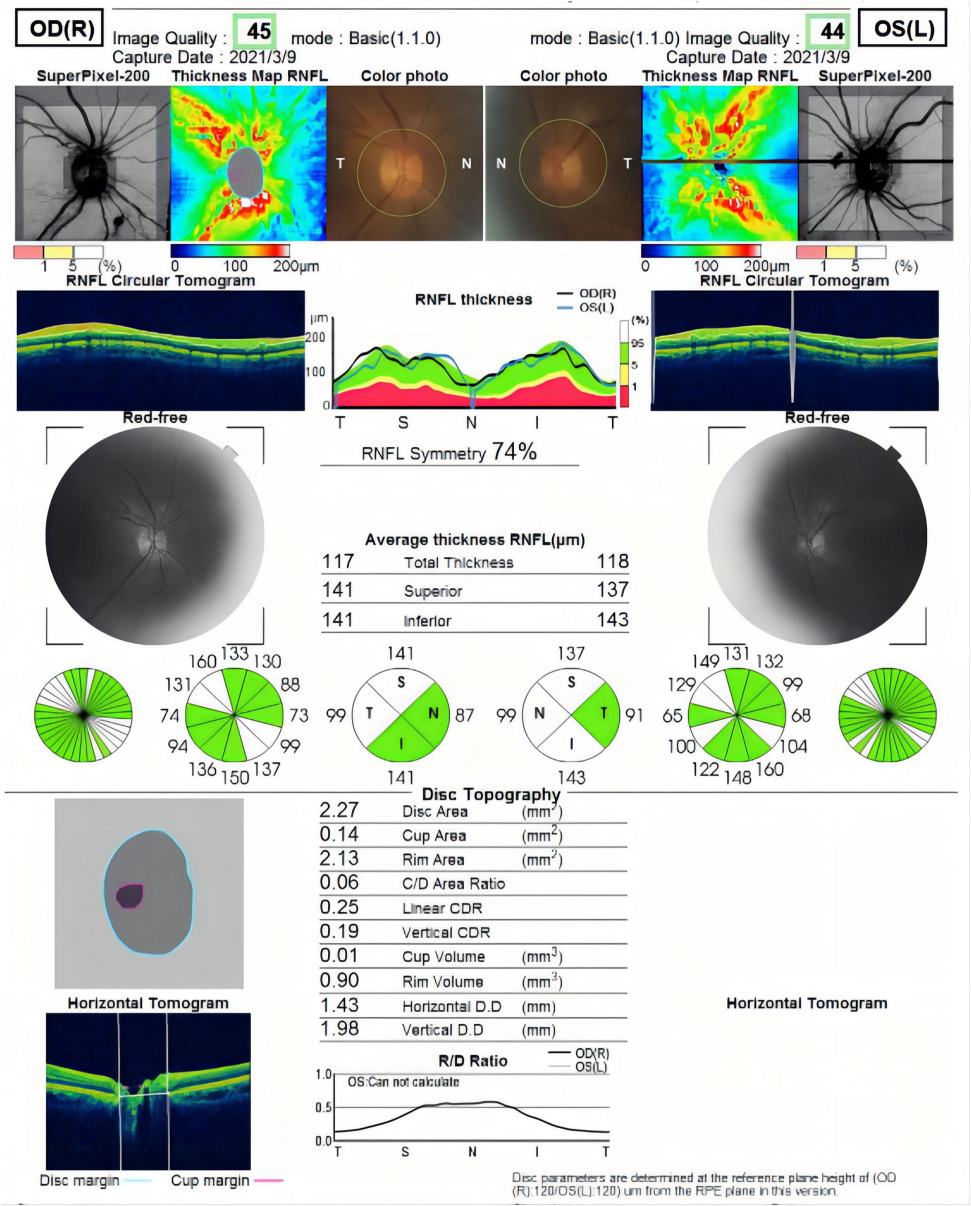
The optical coherence tomography (OCT) examination of retinal nerve fiber layer (RNFL) thickness in the optic disc.

**Figure 4:**
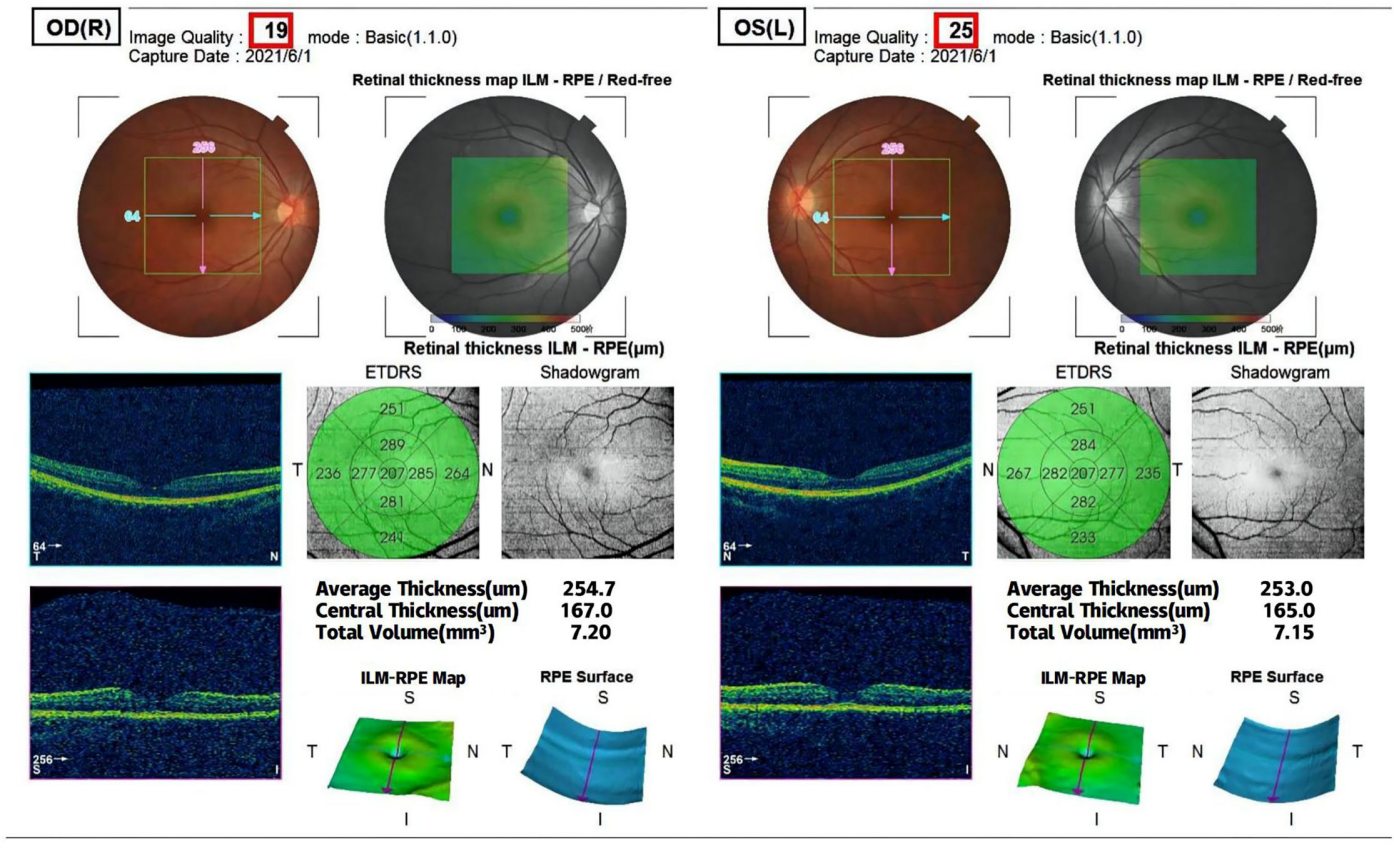
The OCT examination of retinal thickness in the macular region.

All participants received systematic ophthalmic examinations, including slit lamp, intraocular pressure, wide-field fundus photograph (Optos Daytona200, England) (Figure 1), fundus fluorescein angiography (FFA) (Zeiss VISUCAM524, Oberkochen, Germany) (Figure 2), and optical coherence tomography (OCT) (Topcon, Topcon3D-OCT2000, Tokyo, Japan) (Figures 3, 4). Axial length was calculated through IOL MASTER (Zeiss, Oberkochen, Germany).

Image acquisition during OCT was performed by scanning a 6 × 6 mm region around the optic disc and central fovea of the macula. The instrument is spectral domain scanning, and the scanning speed is 54,000/s. Statistical analyses were performed according to the parameters of the upper (s), lower (I), nasal (n), and temporal (T) quadrants. The examination is carried out by professionals. The measurements were done three times and averaged.

### Statistical Methods

All statistical analyses were performed using the SPSS 22.0 software (Chicago, IL, USA). Measurement data were analyzed by the Shapiro–Wilk test. Spherical equivalent, urea, and cholesterol values showed skewed distributions, so the data were log transformed to achieve a normal distribution. The results that met or basically conformed to a normal distribution of measurement data are described as the mean± SD. The mean of the measurement data between two groups was compared with a *t* test, and the F test was applied in comparisons among more than two groups. Normality testing was performed first, followed by Pearson correlation analyses. These tests were used to compare the peripapillary retinal nerve fiber layer (pRNFL) and retina in the macular region and biochemical indexes. Mixed linear models were carried out to identify significant correlations of the pRNFL and macular region values with the final variables obtained in the univariate analysis. Statistics were considered significant at *p* < 0.05.

## Results

This prospective study included 82 eyes in 41 patients (20 women and 21 men), comprising 42 eyes from the prediabetes patient group (10 women and 11 men) and 40 eyes from the healthy control group (10 women and 10 men). The ages of the participants in the healthy control group ranged from 30 to 60 years, with an average age of 46 ± 9.22 years. The ages of the prediabetes patients ranged from 34 to 57 years, with an average age of 44 ± 6.85 years. The mean BCVA (LogMAR) was.02 ± 0.02 and −0.01 ± 0.04 in the healthy control group and prediabetes group, respectively. The mean intraocular pressures (IOPs) were 14.62 ± 3.04 mmHg and 13.07 ± 2.99 mm in the healthy controls and prediabetes patients. The mean diopter values were 1.2 ± 3.18 D and 0.38 ± 1.64 D in the healthy controls and prediabetes patients. The mean axis lengths were 23.22 ± 1.10 mm and 23.07 ± 1.32 mm in the healthy control group and prediabetes group. Prediabetes patients matched in age (*p* = 0.15), gender (*p* = 0.99), BCVA (*p* = 0.09), IOP (*p* = 0.36), spherical equivalent (*p* = 0.24), and axis length (*p* = 0.44) (Table 1) served as healthy controls.

**Table 1:** Baseline characteristics.

**Variable**	**Control (*n* = 20)**	**Prediabetes (*n* = 21)**	***p* value**
Age (years)	46 (9.22)	44(6.85)	0.15
Male gender n (%)	16 (32.0)	55 (36.2)	0.76
BCVA (logMAR)	0.02 (0.02)	−0.01 (0.04)	0.09
IOP–NCT (mmHg)	14.62 (3.04)	13.07 (4.02)	0.36
Spherical equivalent (D)	−1.2 (3.18)	0.38 (1.64)	0.24
Axial length (mm)	23.22 (1.10)	23.07 (1.32)	0.44

*Results are expressed as mean (±SD); n, number of eyes; BCVA, best corrected visual acuity; IOP, intra-ocular pressure; D, diopter*.

*p values were obtained by t tests*.

### Fundus Flfluorescein Angiography (FFA)

Through FFA examination, all patients found no microaneurysm, capillary dilatation, or capillary leakage in microvessels in early, middle, and late stage.

### Analyses of Total PRNFL and Retinal Thickness

**Figure 5:**
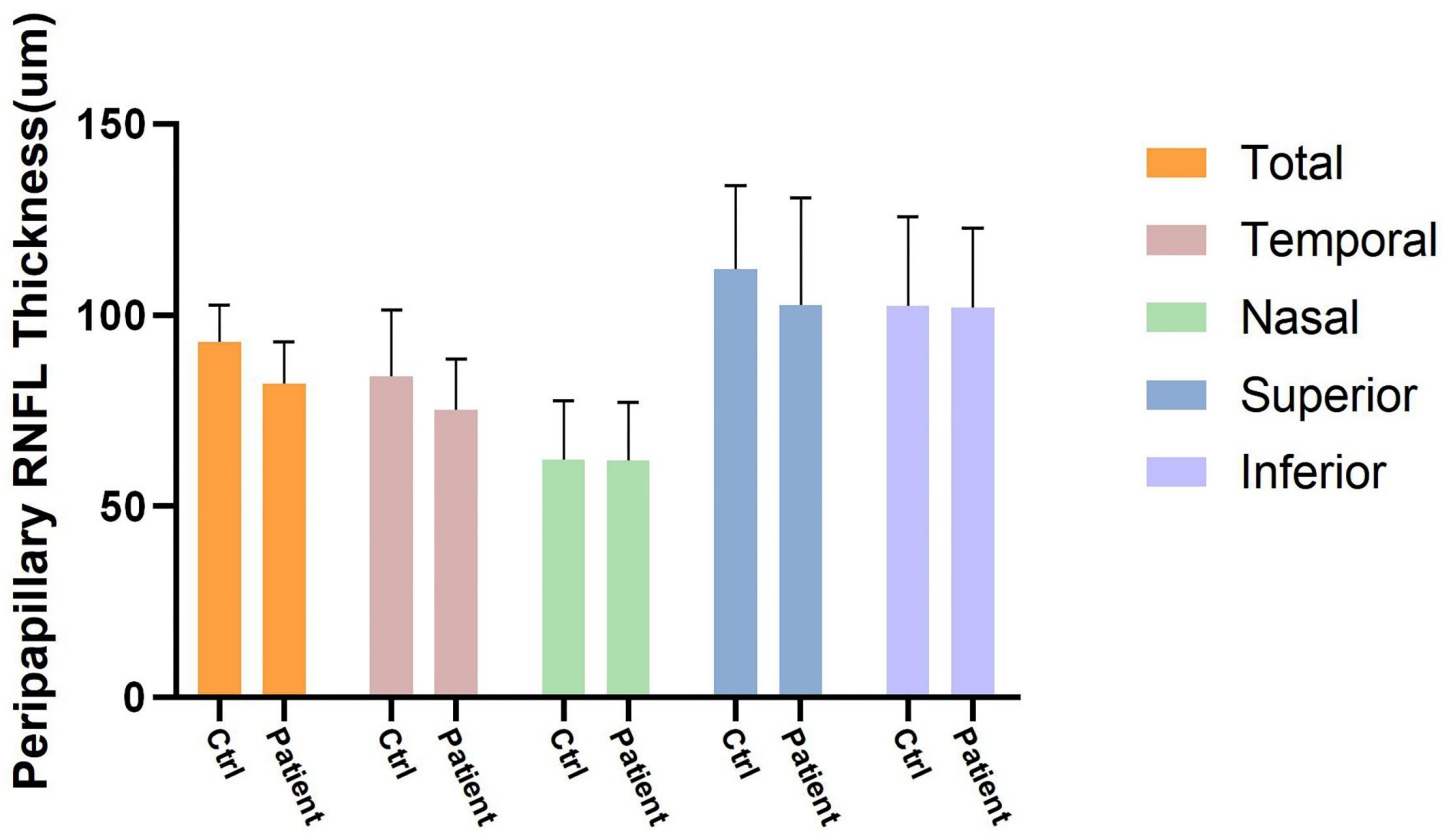
Four quadrants of pRNFL thickness changes (μm) between control group and prediabetes group.

**Figure 6:**
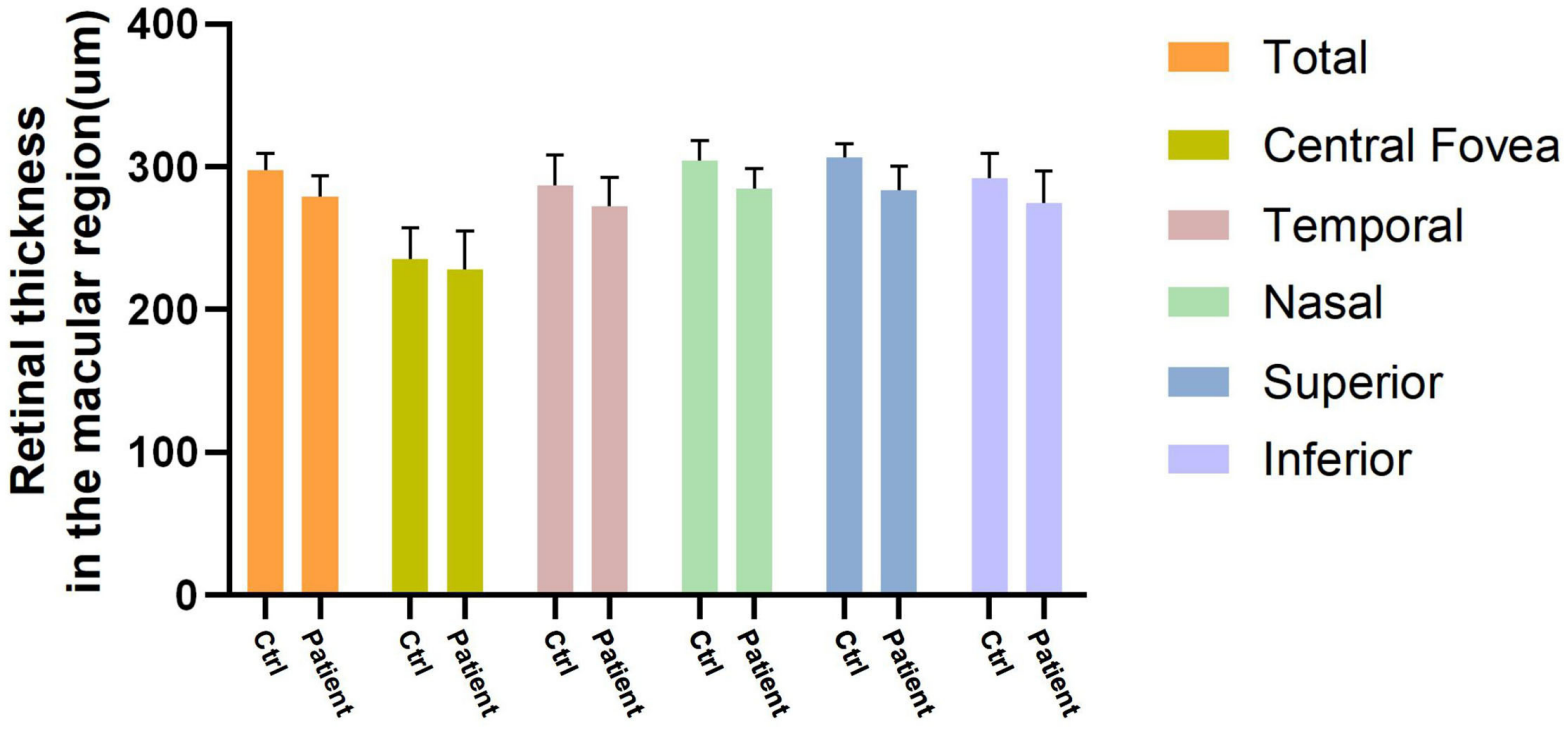
Four quadrants of retinal thickness changes (μm) in the macular region between control group and prediabetes group.

The total pRNFL and retina in macular region in the prediabetes group were significantly thinner than those in the control group (*p* < 0.0001 and *p* < 0.0001, respectively) (Figures 5, 6). The temporal quadrant of the pRNFL was significantly different between the two groups (*p* = 0.011). However, there were no significant differences in the nasal (*p* = 0.934), superior (*p* = 0.092), or inferior (*p* = 0.465) quadrants of the pRNFL. The four quadrants of the retina in the macular region 6 mm around the central fovea, namely, nasal (*p* < 0.0001), superior (*p* < 0.0001), temporal (*p* = 0.008), and inferior (*p* = 0.001), were significantly different between the two groups, although there was no difference in the central fovea (*p* = 0.286) (Table 2).

**Table 2:** Thickness (μm) changes of peripapillary retinal nerve fiber layer (pRNFL) and retina in the macular region compared between control and prediabetes.

**Quadrants**	**Peripapillary RNFL thickness**			**Retinal thickness in the macular region**		
	**Control**	**Prediabetes**	***p* value**	**Control**	**Prediabetes**	***p* value**
**Total**	95.8 (10.3)	80.7 (10.7)	<0.0001	297.3 (11.9)	279.0 (13.8)	<0.0001
**Temporal**	83.3 (15.1)	73.6 (13.9)	0.011	235.0 (23.7)	225.2 (19.0)	0.008
**Nasal**	62.3 (14.6)	62.0 (14.7)	0.934	287.2 (12.2)	272.6 (12.5)	<0.0001
**Superior**	112.2 (22.1)	102.7 (28.6)	0.092	304.8 (10.5)	284.1 (14.4)	<0.0001
**Inferior**	102.5 (23.9)	102.0 (19.7)	0.465	306.3 (16.7)	284.3 (24.2)	0.001
**Central Fovea**	-	-	-	292.3 (17.7)	274.8 (25.3)	0.287

*Image acquisition during optical coherence tomography (OCT) was performed by scanning a 6 × 6 mm region around the optic disc and macula. The central fovea of macular refers to 1 × 1 mm in the macular scanning region. Results are expressed as mean (±SD); p values were obtained by t test*.

**Figure 7:**
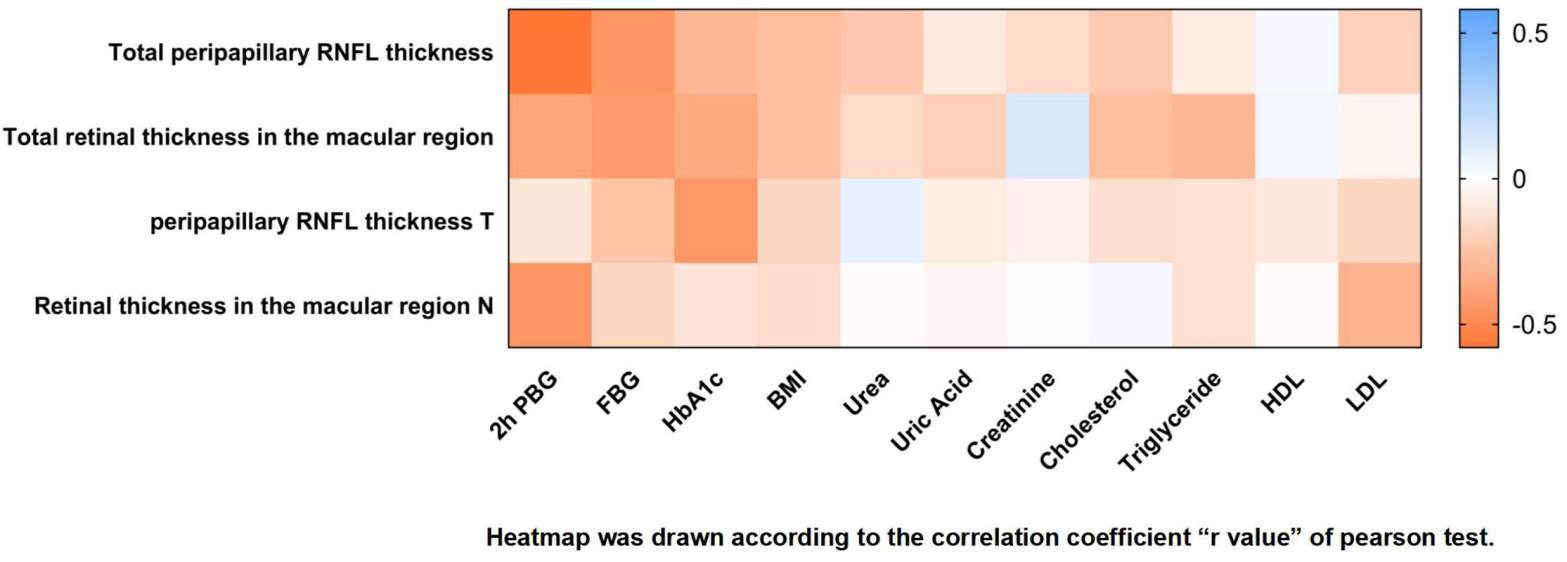
Heat map of correlation analysis of peripapillary RNFL (pRNFL) and retina in the macular region between control group and prediabetes group.

All data were tested by the Shapiro–Wilk test and were found to be normally distributed. Pearson analysis examined the correlation of the pRNFL and retina in the macular region with various laboratory tests. The Pearson correlation coefficient showed a high correlation between total pRNFL and 2h OGTT (r = −0.58; *p* < 0.0001) and FBG (r = −0.45; *p* = 0.0040). The variables with smaller correlations were BMI (r = −0.28; *p* = 0.0448), HbA1c (r = −0.31; *p* = 0.0391), urea (r = −0.24; *p* = 0.0454), and cholesterol (r = −0.23; *p* = 0.0472). Moreover, the variable that showed a high correlation with the retina in the macular region thickness was FBG (r = −0.43; *p* = 0.0056). The variables with smaller correlations were the 2 h OGTT (r = −0.38; *p* = 0.0158) and HbA1c (r = −0.37; *p* = 0.0222). The variable associated with the temporal quadrant pRNFL was HbA1c (r = −0.44; *p* = 0.0049), and the variable associated with the nasal quadrant of the retina in the macular region was the 2 h OGTT (r = −0.45; *p* = 0.0041) (Table 3 and Figure 7).

**Table 3:** Pearson correlation analysis of pRNFL and retina in the macular region influence factors.

**Variable**	**Total pRNFL thickness**		**Total retinal thickness in the macular region**		**pRNFL thickness T**		**Retinal thickness in the macular region N**	
	***p* value**	**r**	***p* value**	**r**	***p* value**	**r**	***p* value**	**r**
2h OGTT	0.0001	−0.58	0.0158	−0.38	0.5037	−0.11	0.0041	−0.45
FBG	0.0040	−0.45	0.0056	−0.43	0.1138	−0.26	0.2941	−0.17
HbA1c	0.0391	−0.31	0.0222	−0.37	0.0049	−0.44	0.4492	−0.12
BMI	0.0448	−0.28	0.0913	−0.27	0.2976	−0.17	0.3410	−0.15
Urea	0.0454	−0.24	0.2899	−0.16	0.6899	0.08	0.9355	−0.02
Uric Acid	0.2733	−0.09	0.1439	−0.20	0.6992	−0.08	0.8421	−0.03
Creatinine	0.0783	−0.16	0.3599	0.13	0.8397	−0.06	0.9772	0.01
Cholesterol	0.0472	−0.23	0.0863	−0.28	0.3803	−0.14	0.8263	0.03
Triglyceride	0.3522	−0.08	0.0772	−0.31	0.3122	−0.13	0.4098	−0.13
HDL	0.5347	0.03	0.5627	0.04	0.5799	−0.10	0.9289	−0.02
LDL	0.0562	−0.20	0.5238	−0.04	0.2838	−0.17	0.0938	−0.33

**Figure 8:**
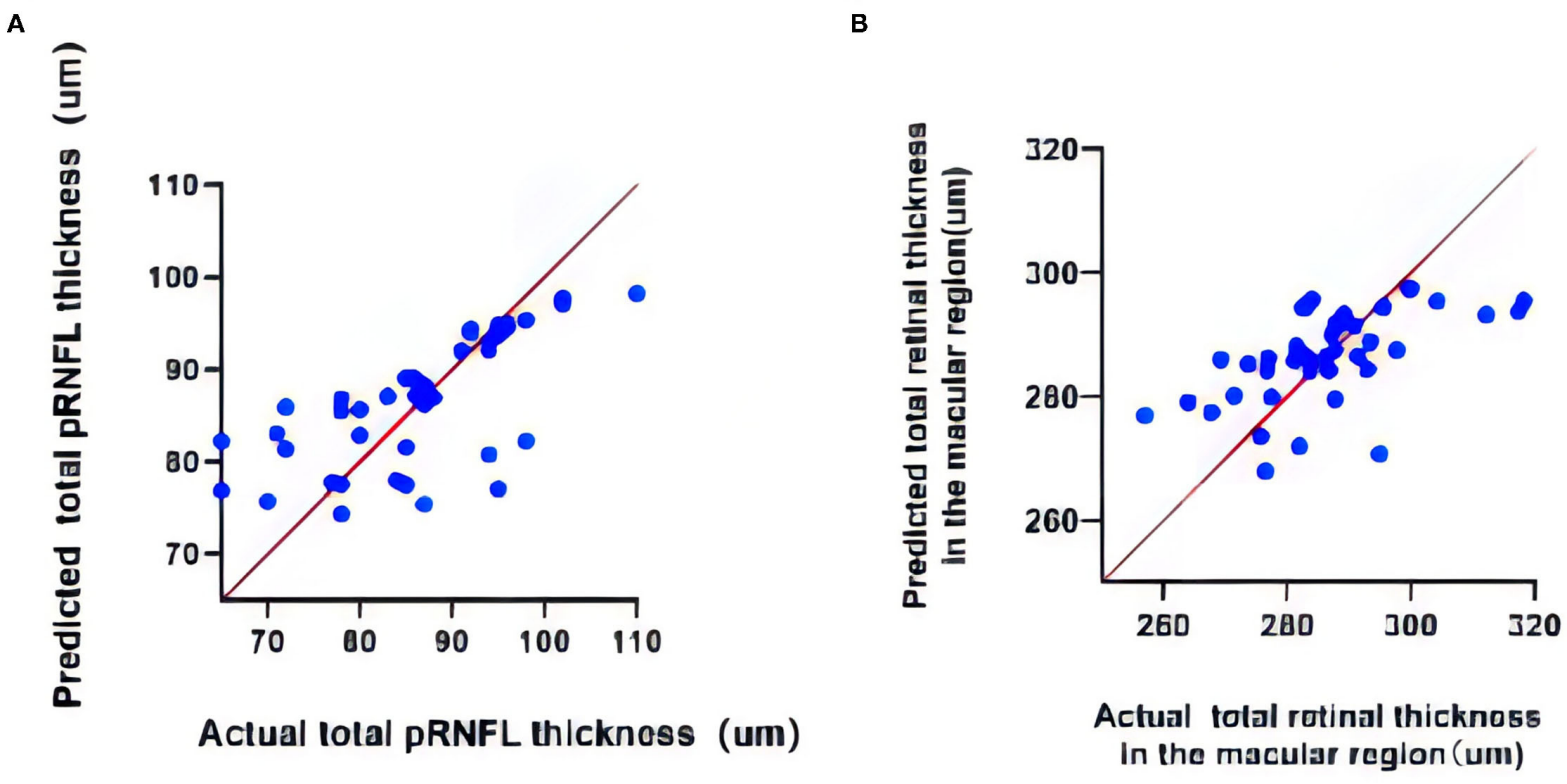
**(A)** The multivariable regression analysis of total pRNFL thickness. **(B)** The multivariable regression analysis of total retinal thickness in the macular region.

In the mixed linear analyses, thickness of pRNFL and retina in the macular region were taken as fixed effect variables, while 2 h OGTT and FBG were taken as covariates. Two-hour OGTT and FBG showed powerful predictabilities to the pRNFL, but poor predictabilities to the retina in the macular region (Figures 8A,B). The mean prediction equation: pRNFL thickness = 139.3–3.886^*^2 hOGTT-4.467^*^FBG (*p* < 0.0001), coefficient estimates are shown in the table below (Table 4). It can be inferred that the poor control of 2 h OGTT and FBG causes damage to pRNFL, and that the pRNFL becomes thinner with the increase of 2 h OGTT and FBG.

**Table 4:** Results of multivariable regression models-dependent variable.

**Model**	**Coefficient estimate**	***p* value**	**95% Confidence interval**
**Dependent variable: Total peripapillary RNFL thickness**			
Prediabetes	139.3	<0.0001	120.4 to 158.3
2h OGTT	−3.886	0.0011	−6.107 to −1.665
FBG	−4.467	0.0191	−8.159 to −0.7742
**Dependent variable: Total retinal thickness in the macular region**			
Prediabetes	334.3	<0.0001	307.9 to 360.7
2h OGTT	−5.811	0.0005	−8.896 to −2.725
FBG	−0.5042	0.8434	−5.634 to 4.626

*Reference category: control group; p values were obtained by linear regression models*.

## Discussion

The main finding of our study was that none of the prediabetic patients had significant abnormalities on microvascular examination compared with normal age- and sex-matched subjects. However, both the pRNFL thickness and nasal macular thickness were reduced in the temporal quadrant, and the correlations between these changes and other systemic indexes were further analyzed.

Prediabetes is defined as an abnormal state of glucose homeostasis in which blood glucose levels are higher than normal but not as high as those required for a diagnosis of diabetes and is an intermediate state between normal glucose homeostasis and the pathological state of diabetes (3). Internationally, prediabetes criteria include IFG, IGT, and HbA1c (IA1c) (4). However, in China, guidelines have not yet recommended the adoption of HbA1c as a diagnostic criterion for diabetes due to insufficient standardization of HbA1c testing methods and different HbA1c diagnostic cutoff points in different regions (5).

Fundus Fluorescein Angiography (FFA) is the gold standard for observing retinal microvascular damage. No microangiomas were observed in any of the preclinical patients with diabetes in our observations (6). An increasing number of studies have analyzed the retinal nerve fiber layer (RNFL) by noninvasive search methods, such as OCT, which is an important tool for the early detection of retinal nerve changes. Most previous studies, including ours (2), showed thinning of the RNFL and thinning of the macula in patients with diabetes mellitus (DM), although there is no exact agreement between studies regarding which quadrants of the peripapillary RNFL and macular thickness is affected. Irini Chatziralli found that peripapillary RNFL thickness was reduced in patients with DM in all quadrants (7). Dhasmana R observed RNFL thinning in the superotemporal and superior nasal regions around the optic disc in eyes with DR (8). Another study on patients with type 1 DM without DR showed RNFL thinning only in the superior quadrant (9). Garcia-Martin E et al. observed a decrease in mean peripapillary RNFL thickness, inferior thickness, and infratemporal thickness (10). We speculate that the study variability in the population and the different imaging modalities and procedures used to measure the peripapillary RNFL may have led to variability in the results. However, all previous studies have focused on controlled observations in diabetic patients with diabetic retinopathy, diabetic patients without diabetic retinopathy, and normal populations, and there is a lack of studies on prediabetes. We evaluated the neurological changes around the optic disc and macula using the scanning method described in the methods section above and found thinning in both the temporal RNFL around the optic papilla and the nasal retinal thickness in the macula.

We believe that the above characteristics of nerve damage correlate with the distribution of retinal nerve fiber layers. The thickness of the RNFL varied in each region. In healthy populations, the RNFL is thickest on the temporal side of the upper quadrant (11) and is especially dense in the temporal arch (12). Our observations of thinning of the RNFL on the temporal side of the optic papilla correspond to changes in retinal thickness on the nasal side of the macula, suggesting that the injury first appears in the area of the macular bundle of the papilla where the nerve fibers are most densely distributed. We speculate that this may be related to the physical density and oxygen demand of the dense alignment of nerve fibers and thus to a direct correlation between sensitivity and susceptibility to injury from sugar fluctuations. Previous studies have shown that the retinal capillary basement membrane is thickened in diabetic retinopathy, resulting in reduced oxygen diffusion from the capillaries to the tissue. The hyperglycemia-induced retinal changes are a reduction in tissue area and oxygen consumption, resulting in increased retinal arterial oxygen saturation (13). Temporal retinal oxygen saturation was found to be higher in early diabetic patients than in healthy controls (14). Previous studies have shown that patients with prediabetes are at similarly high risk for a range of neurological complications (15). Recently, the MONICA/KORA study showed that IGT neuropathy (neuropathic pain score) was more common in patients than in controls (16). Lee et al. also found that prediabetes was independently associated with the presence of peripheral neuropathy and the severity of neurological dysfunction (17). These results are consistent with our findings.

A potential limitation of our cross-sectional study is the small number of participants. Studies with longitudinal follow-up may better detect the effects of peak and duration of prediabetic glucose levels on retinal nerves and blood vessels. In addition, we analyzed data from both eyes of the enrolled individuals, which may underestimate standard errors and lead to low p values.

Our findings suggest that minor abnormalities in the retinal nerves, where no clear microvascular abnormalities were observed in the retina, had developed in prediabetes. Thus, lesions of the retinal nerve in prediabetes may precede microvascular changes. Glycemic control is important in preventing the development of retinal nerve complications, while neuroprotective therapy may also be important in the management of prediabetic ocular complications.

## Data Availability Statement

The raw data supporting the conclusions of this article will be made available by the authors, without undue reservation.

## Ethics Statement

The studies involving human participants were reviewed and approved by the Ethics Committee of the Third Affiliated Hospital of Sun Yat-sen University. The patients/participants provided their written informed consent to participate in this study.

## Author Contributions

All authors listed have made a substantial, direct, and intellectual contribution to the work and approved it for publication.

## Conflict of Interest

The authors declare that the research was conducted in the absence of any commercial or financial relationships that could be construed as a potential conflict of interest. The handling Editor declared a shared affiliation with the authors at time of review.

## Publisher's Note

All claims expressed in this article are solely those of the authors and do not necessarily represent those of their affiliated organizations, or those of the publisher, the editors and the reviewers. Any product that may be evaluated in this article, or claim that may be made by its manufacturer, is not guaranteed or endorsed by the publisher.
